# Early prediction of noninvasive ventilation failure in COPD patients: derivation, internal validation, and external validation of a simple risk score

**DOI:** 10.1186/s13613-019-0585-9

**Published:** 2019-09-30

**Authors:** Jun Duan, Shengyu Wang, Ping Liu, Xiaoli Han, Yao Tian, Fan Gao, Jing Zhou, Junhuan Mou, Qian Qin, Jingrong Yu, Linfu Bai, Lintong Zhou, Rui Zhang

**Affiliations:** 1 Department of Respiratory and Critical Care Medicine, The First Affiliated Hospital of Chongqing Medical University, Youyi Road 1, Yuzhong District, Chongqing, 400016 People’s Republic of China; 2 Department of Pulmonary and Critical Care Medicine, The First Affiliated Hospital of Xi’an Medical University, Xi’an, 710077 People’s Republic of China; 3 Department of Respiratory and Critical Care Medicine, The People’s Hospital of Changshou, Chongqing, 401220 People’s Republic of China

**Keywords:** COPD exacerbations, Noninvasive ventilation, Critical care

## Abstract

**Background:**

Early identification of noninvasive ventilation (NIV) failure is a promising strategy for reducing mortality in chronic obstructive pulmonary disease (COPD) patients. However, a risk-scoring system is lacking.

**Methods:**

To develop a scale to predict NIV failure, 500 COPD patients were enrolled in a derivation cohort. Heart rate, acidosis (assessed by pH), consciousness (assessed by Glasgow coma score), oxygenation, and respiratory rate (HACOR) were entered into the scoring system. Another two groups of 323 and 395 patients were enrolled to internally and externally validate the scale, respectively. NIV failure was defined as intubation or death during NIV.

**Results:**

Using HACOR score collected at 1–2 h of NIV to predict NIV failure, the area under the receiver operating characteristic curves (AUC) was 0.90, 0.89, and 0.71 for the derivation, internal-validation, and external-validation cohorts, respectively. For the prediction of early NIV failure in these three cohorts, the AUC was 0.91, 0.96, and 0.83, respectively. In all patients with HACOR score > 5, the NIV failure rate was 50.2%. In these patients, early intubation (< 48 h) was associated with decreased hospital mortality (unadjusted odds ratio = 0.15, 95% confidence interval 0.05–0.39, *p* < 0.01).

**Conclusions:**

HACOR scores exhibited good predictive power for NIV failure in COPD patients, particularly for the prediction of early NIV failure (< 48 h). In high-risk patients, early intubation was associated with decreased hospital mortality.

## Background

Noninvasive ventilation (NIV) increases alveolar ventilation and reduces the work of breathing in patients with acute exacerbation of chronic obstructive pulmonary disease (COPD) [1]. Consequently, it reduces respiratory rate, decreases PaCO_2_ and improves the level of consciousness [2, 3]. However, the failure rate ranges from 15 to 24% in COPD patients [4–6]. In contrast to patients who initially receive invasive mechanical ventilation, patients who initially receive NIV but subsequently experience NIV failure and then receive intubation are more likely to die in the hospital [7, 8]. And delayed intubation is associated with increased hospital mortality [9–11]. Thus, the early identification of patients who cannot benefit from NIV is important.

Previous studies have reported that many variables can predict NIV failure in COPD patients [5, 12–16], including disease severity, heart rate, respiratory rate, consciousness, and arterial blood pH. However, no single variable can predict NIV failure well. A combination of several variables may increase predictive accuracy. Thus, we developed a simple score using several variables that are easily obtained at bedside to assess the efficacy of NIV in COPD patients.

## Methods

This study was performed at three hospitals (the First Affiliated Hospital of Chongqing Medical University, the First Affiliated Hospital of Xi’an Medical University, and the People’s Hospital of Changshou). At the First Affiliated Hospital of Chongqing Medical University, data were extracted from a prospectively collected database which was collected from June 2011 to June 2018. We randomly selected 500 patients to the derivation cohort and the rest 323 patients to the internal-validation cohort. At the First Affiliated Hospital of Xi’an Medical University and the People’s Hospital of Changshou, data were retrospectively collected from January 2015 to June 2018 and March 2013 to June 2018, respectively. These data were from a total of 395 patients, who were enrolled in an external-validation cohort.

The ethics committee approved the study protocol. Informed consent was obtained from patients or their next of kin at the First Affiliated Hospital of Chongqing Medical University. As data were retrospectively collected at the other two hospitals, informed consent was waived. We enrolled COPD patients admitted to the respiratory ICU for NIV as follows: arterial blood pH less than 7.35, PaCO_2_ more than 45 mmHg, and presence of dyspnea at rest assessed using accessory respiratory muscles or paradoxical abdominal breathing. However, patients who required emergency intubation or showed NIV intolerance were excluded. NIV intolerance was defined as refusal of NIV because of discomfort [17].

Patients were managed by the attending physicians, respiratory therapists, and nurses. The NIV was managed according to the protocols of each hospital. The face mask was the first choice for the interface to connect the ventilator to the patient. The size of the face mask was fitted to the face type. Bi-level positive airway pressure was used for the ventilation mode. The expiratory positive airway pressure was initially set at 4 cmH_2_O, and it was titrated to diminish the ineffective trigger. The inspiratory positive airway pressure was initially set at 8 cmH_2_O and then gradually increased to achieve the best control of dyspnea or to the tolerance of the patient. The fractional concentration of oxygen was selected to maintain the bedside oximeter (SpO_2_) above 90% and the PaO_2_ above 60 mmHg.

The efficacy of NIV was assessed during NIV intervention. The arterial blood pH, PaO_2_, PaCO_2_, PaO_2_/FiO_2_, GCS, respiratory rate, and heart rate were recorded. If respiratory failure was relieved, liberation from NIV was attempted. However, if the conditions of respiratory failure became progressively aggravated and reached the criteria for invasive mechanical ventilation, intubation was performed. The criteria for intubation were persistent respiratory distress with respiratory rate more than 35 breaths/min, failure to maintain a PaO_2_/FiO_2_ above 100 mmHg, inability to correct respiratory acidosis, development of conditions necessitating intubation to protect the airway (coma or seizure disorders) or to manage copious tracheal secretions, hemodynamic instability without response to fluids and vasoactive agents, and respiratory or cardiac arrest. Nevertheless, intubation was left to physician discretion. NIV failure was defined as intubation or death during NIV [5]. Where NIV failure occurred within 48 h of NIV, it was defined as early NIV failure [18]. Where failure occurred after 48 h of NIV, it was defined as late NIV failure.

### Statistical analysis

Data for 24 patients (2%) were unavailable for at least one time point. No imputation for missing data was performed. Data were analyzed using statistical software (SPSS 17.0; IBM Corp., Armonk, NY). Qualitative and categorical variables are reported as numbers and percentages, and the differences among groups were analyzed using the Chi-square and/or Fisher’s exact tests. The differences between pairs of groups were analyzed using the unpaired Student’s *t*-test or the Mann–Whitney *U* test, where appropriate. The differences among the three groups were analyzed using one-way ANOVA or the Kruskal–Wallis *H* test, where appropriate. The diagnostic accuracy of NIV failure was analyzed using area under the receiver operating characteristic curves (AUC). The cutoff value was based on a positive likelihood ratio of NIV failure of > 5 [19]. Differences that exhibited *p* values less than 0.05 were considered statistically significant.

The risk-scoring system was developed through the following steps. First, univariate analyses were used to identify variables associated with NIV failure collected at initiation and 1–2 h of NIV in the derivation cohort. Second, variables with *p* values less than 0.1 in univariate analyses were entered in stepwise multivariate logistic regression analyses to identify the independent risk factors associated with NIV failure. However, we omitted the heart rate collected before NIV as it was strongly correlated with that collected at 1–2 h of NIV and the ability to predict NIV failure was lower than that collected at 1–2 h of NIV [20]. At this step, a regression model was obtained to predict NIV failure. We evaluated this model for goodness of fit using the Hosmer–Lemeshow test (*p* > 0.05). Third, the risk-scoring system was created, following the method suggested by Sullivan et al. [21]. The variables in the final model were classified into clinically meaningful categories, and the median for each category was recorded. For each variable, a category bearing the lowest risk for NIV failure was set as the within-group reference, and it was assigned to the zero point. Each category was weighted (the difference between reference category multiplied by the *β* regression coefficient per unit increase). Then, one point was assigned to the category with the lowest weight, and this weight was set as the between-group reference. Finally, we calculated the value of that weight for the other category, divided by the between-group reference. This value was rounded off to the nearest integer as to create the assigned points. The sum of the points was the risk score.

## Results

The NIV failure rate was 18.8%, 18.9% and 8.9% in derivation, internal-validation and external-validation cohorts, respectively (Additional file 1: Figure S1). The demographics of each cohort are summarized in Table 1. In the derivation cohort, we found that 14 variables collected at initiation and 1–2 h of NIV were associated with NIV failure in univariate analyses (Table 2). However, the heart rate, acidosis (assessed by pH), consciousness (assessed using the GCS), oxygenation, and respiratory rate were each independently associated with NIV failure. These five variables were used to develop a risk-scoring system to predict NIV failure. Following the weights for each variable, we assigned 3 points to heart rate, 8 points to acidosis, 11 points to consciousness, 2 points to oxygenation, and 3 points to respiratory rate (Table 3). We called the result the HACOR score, on a scale of a total of 27 points.

**Table 1:** Demographics among the derivation, internal-validation, and external-validation cohorts

	Derivation cohort *N* = 500	Internal-validation cohort *N* = 323	External-validation cohort *N* = 395	*p*
Age, years	71.9 ± 10.1	72.2 ± 10.1	73.1 ± 9.7	0.16
Male	371 (74.2%)	255 (78.9%)	266 (67.3%)	< 0.01*
APACHE II score	17.1 ± 4.9	17.2 ± 5.0	13.5 ± 5.2	< 0.01*
Underlying disease				
Diabetes mellitus	92 (18.4%)	53 (16.4%)	26 (6.6%)	< 0.01*
Hypertension	190 (38.0%)	126 (39.0%)	119 (30.1%)	0.02*
Chronic heart disease	139 (27.8%)	84 (26.0%)	131 (33.2%)	0.08
Chronic kidney disease	47 (9.4%)	22 (6.8%)	13 (3.4%)	< 0.01*
Chronic liver disease	35 (7.0%)	31 (9.6%)	1 (0.3%)	< 0.01*
Data collected before NIV				
Respiratory rate, breaths/min	28 ± 6	29 ± 6	24 ± 4	< 0.01*
Heart rate, beats/min	109 ± 21	110 ± 21	103 ± 22	< 0.01*
Systolic blood pressure, mmHg	139 ± 27	141 ± 25	134 ± 24	< 0.01*
Diastolic blood pressure, mmHg	81 ± 16	83 ± 17	77 ± 15	< 0.01*
pH	7.26 ± 0.07	7.27 ± 0.06	7.25 ± 0.08	0.02*
PaCO_2_, mmHg	82 ± 18	81 ± 17	79 ± 19	0.14
PaO_2_/FiO_2_, mmHg	197 ± 94	208 ± 108	217 ± 112	0.02*
GCS	14.4 ± 1.5	14.4 ± 1.5	14.0 ± 2.4	< 0.01*
Data collected at 1–2 h of NIV				
Respiratory rate, breaths/min	23 ± 5	24 ± 5	22 ± 4	< 0.01*
Heart rate, beats/min	100 ± 21	100 ± 20	96 ± 21	0.01*
Systolic blood pressure, mmHg	126 ± 23	128 ± 20	125 ± 20	0.26
Diastolic blood pressure, mmHg	73 ± 14	74 ± 13	72 ± 12	0.47
Minute ventilation, L/min	7.9 ± 3.2	8.0 ± 3.4	8.4 ± 2.9	0.03*
IPAP, cmH_2_O	17 ± 4	17 ± 4	17 ± 4	0.44
EPAP, cmH_2_O	6 ± 2	6 ± 2	5 ± 1	< 0.01*
pH	7.33 ± 0.08	7.33 ± 0.08	7.31 ± 0.08	0.01*
PaCO_2_, mmHg	72 ± 18	72 ± 18	72 ± 19	0.81
PaO_2_/FiO_2_, mmHg	220 ± 89	222 ± 84	201 ± 81	< 0.01*
GCS	14.5 ± 1.5	14.5 ± 1.4	14.2 ± 2.1	0.04*
Outcomes				
NIV failure	94 (18.8%)	61 (18.9%)	35 (8.9%)	< 0.01*
Early NIV failure	64 (12.8%)	33 (10.2%)	18 (4.6%)	< 0.01*
Late NIV failure	30 (6.0%)	28 (8.7%)	17 (4.3%)	0.05
ICU mortality	57 (11.4%)	39 (12.1%)	15 (3.8%)	< 0.01*
Hospital mortality	66 (13.2%)	47 (14.6%)	23 (5.8%)	< 0.01*

*GCS* Glasgow coma scale, *NIV* noninvasive ventilation, *IPAP* inspiratory positive airway pressure, *EPAP* expiratory positive airway pressure, *ICU* intensive care unit

*p < 0.05 between three groups

**Table 2:** Univariate and multivariate analyses for risk factors associated with NIV failure in the derivation cohort

	Univariate analyses OR (95% CI)	*p*	Multivariate analyses OR (95% CI)	*p*
Chronic kidney disease	2.219 (1.147–4.292)	0.02	–	–
APACHE II score	1.208 (1.150–1.270)	< 0.01	–	–
Data collected before NIV				
Heart rate, beats/min	1.025 (1.014–1.037)	< 0.01	–	–
pH	0.004 (0.000–0.084)	< 0.01	–	–
PaO_2_/FiO_2_, mmHg	0.996 (0.993–0.999)	0.01	–	–
GCS	0.651 (0.558–0.759)	< 0.01	–	–
Data collected at 1–2 h of NIV				
Respiratory rate, breaths/min	1.122 (1.076–1.170)	< 0.01	1.064 (1.001–1.131)	0.05
Heart rate, beats/min	1.035 (1.024–1.047)	< 0.01	1.027 (1.011–1.043)	< 0.01
Systolic blood pressure, mmHg	1.015 (1.005–1.024)	< 0.01	–	–
Diastolic blood pressure, mmHg	1.027 (1.011–1.044)	< 0.01	–	–
pH	0.000 (0.000–0.000)	< 0.01	0.000 (0.000–0.000)	< 0.01
PaCO_2_, mmHg	1.037 (1.024–1.051)	< 0.01	–	–
PaO_2_/FiO_2_, mmHg	0.991 (0.987–0.994)	< 0.01	0.995 (0.990–0.999)	0.02
GCS	0.331 (0.245–0.447)	< 0.01	0.371 (0.269–0.512)	< 0.01

*NIV* noninvasive ventilation, *OR* odds ratio, *CI* confidence interval, *GCS* Glasgow coma scale

**Table 3:** Final model for prediction of NIV failure in the derivation cohort and points assigned to each variable

Variables	Regression coefficient *β* per unit increase	Category (*j*)	Assigned points
Respiratory rate at 1–2 h of NIV, breaths/min	0.062	< 30	0
		30–34	1
		35–39	2
		≥ 40	3
Heart rate at 1–2 h of NIV, beats/min	0.027	< 100	0
		100–119	1
		120–139	2
		≥ 140	3
pH at 1–2 h of NIV	− 15.851	≥ 7.35	0
		7.30–7.34	2
		7.25–7.29	3
		7.20–7.24	5
		< 7.20	8
GCS at 1–2 h of NIV	− 0.992	15	0
		14	2
		13	4
		12	6
		≤ 11	11
PaO_2_/FiO_2_ at 1–2 h of NIV, mmHg	− 0.005	≥ 150	0
		101–149	1
		≤ 100	2

*NIV* noninvasive ventilation, *GCS* Glasgow coma scale

**Fig. 1:**
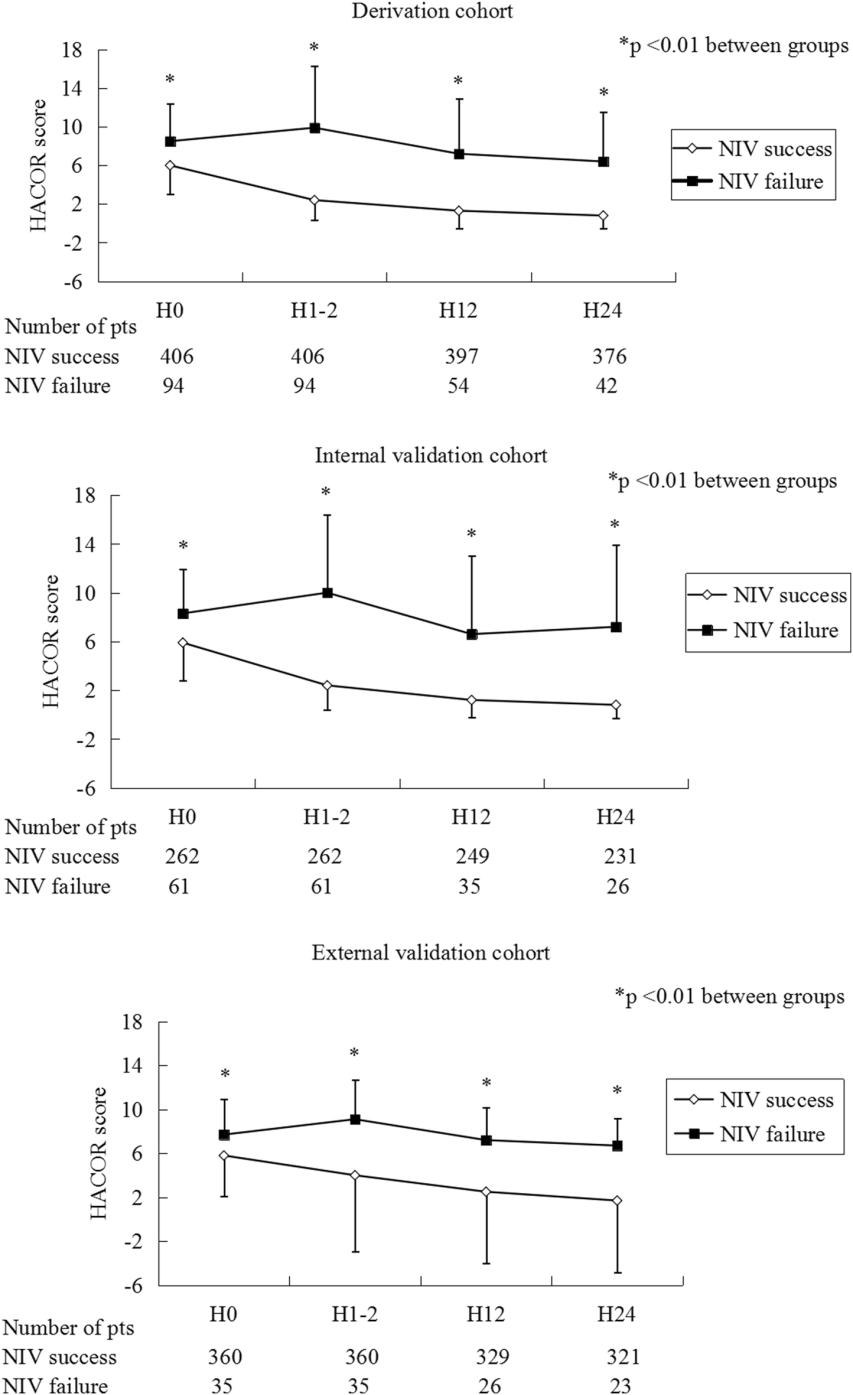
HACOR scores in patients with NIV success and failure from initiation to 24 h of NIV. *Pts* patients, *NIV* noninvasive ventilation, *HACOR* heart rate, acidosis, consciousness, oxygenation, and respiratory rate

**Fig. 2:**
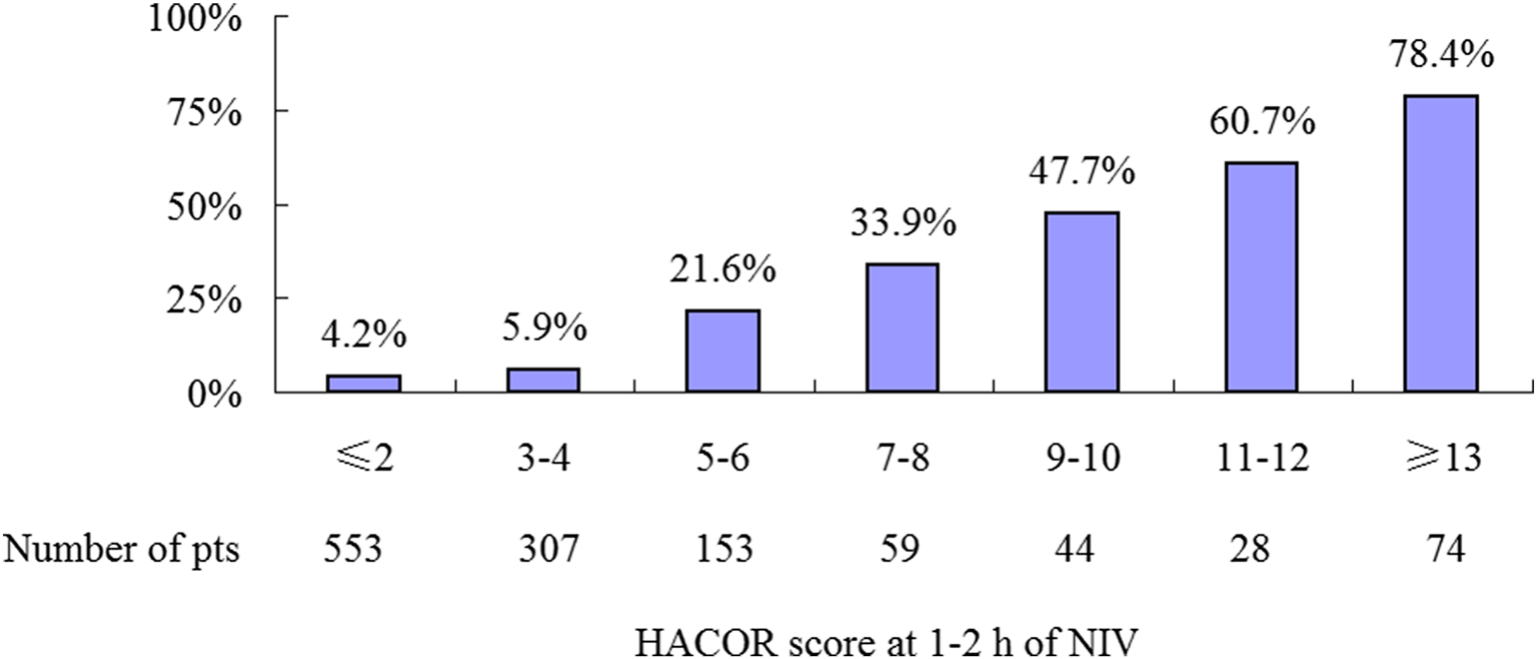
NIV failure rates in patients with different HACOR scores. *Pts* patients, *NIV* noninvasive ventilation, *HACOR* heart rate, acidosis, consciousness, oxygenation, and respiratory rate

In the derivation, internal-validation, and external-validation cohorts, HACOR scores were much lower in the NIV-success patients than the NIV-failure ones at initiation, 1–2 h, 12 h, and 24 h of NIV (Fig. 1). Higher HACOR scores were associated with increased NIV failure (Fig. 2).

At 1–2 h of NIV, the AUC for the prediction of NIV failure was 0.90 and 0.89 in the derivation and internal-validation cohorts, respectively (Table 4). Based on timeframe of admission, we divided the patients into four groups to internally validate the HACOR score once again (Additional file 2: Table S1). The AUC for the prediction of NIV failure was 0.87, 0.89, 0.93, and 0.86 in patients enrolled from 2011 to 2012, 2013 to 2014, 2015 to 2016, and 2017 to 2018, respectively. In external-validation cohort, the AUC for prediction of NIV failure was 0.71 (Table 4). For the prediction of early NIV failure, the AUC was 0.91, 0.96, and 0.83 in the derivation, internal-validation, and external-validation cohorts, respectively. Comparisons in AUC among derivation, internal-validation, and external-validation cohorts are summarized in Additional file 3: Table S2. Comparison in AUC between HACOR score and risk chart developed by Confalonieri et al. [5] to predict NIV failure in external-validation cohort is summarized in Additional file 4: Figure S2.

**Table 4:** Predictive power of NIV failure identified by HACOR score at 1–2 h of NIV

	Cutoff point	AUC (95% CI)	SE (%)	SP (%)	PPV (%)	NPV (%)	LR+	LR−
Derivation cohort								
Prediction of NIV failure	> 5	0.90 (0.87–0.92)	70.2	92.6	68.8	93.1	9.50	0.32
Prediction of early NIV failure	> 5	0.91 (0.88–0.93)	81.3	89.9	54.2	97.0	8.05	0.21
Internal-validation cohort								
Prediction of NIV failure	> 5	0.89 (0.85–0.92)	77.1	93.5	73.4	94.6	11.9	0.25
Prediction of early NIV failure	> 5	0.96 (0.94–0.98)	93.9	88.6	48.4	99.2	8.26	0.07
External-validation cohort								
Prediction of NIV failure	> 5	0.71 (0.67–0.76)	62.9	75.8	20.2	95.5	2.60	0.49
Prediction of early NIV failure	> 5	0.83 (0.79–0.87)	77.8	74.8	12.8	98.6	3.09	0.30

*HACOR* heart rate, acidosis, consciousness, oxygenation and respiratory rate, *NIV* noninvasive ventilation, *AUC* area under the curve of receiver operating characteristics, *CI* confidence interval, *SE* sensitivity, *SP* specificity, *PPV* positive predictive value, *NPV* negative predictive value, *LR+* positive likelihood ratio, *LR−* negative likelihood ratio

A cut-off value of 5 was identified to distinguish the high or low risk of NIV failure assessed by HACOR score at 1–2 h of NIV (Table 4, Additional file 5: Table S3 and Additional file 6: Table S4). In patients with HACOR score ≤ 5, the NIV failure rate was 5.8% (Additional file 7: Figure S3). However, in those with HACOR score > 5, the NIV failure rate was 50.2%. At beginning of NIV, there were no differences between early and late intubation in patients at high risk of NIV failure (Table 5). However, early intubation was associated with decreased hospital mortality (unadjusted odds ratio = 0.15, 95% confidence interval 0.05–0.39, *p* < 0.01).

**Table 5:** Early versus late intubation in patients with HACOR score > 5 at 1–2 h of NIV

	Early intubation *N* = 92	Late intubation *N* = 29	*p*
Age, years	72.5 ± 9.8	76.0 ± 9.1	0.09
Male	73 (79.3%)	22 (75.9%)	0.80
APACHE II score	21.4 ± 5.4	22.3 ± 5.9	0.45
Underlying disease			
Diabetes mellitus	16 (17.4%)	4 (13.8%)	0.78
Hypertension	34 (37.0%)	13 (44.8%)	0.51
Chronic heart disease	23 (25.0%)	8 (27.6%)	0.81
Chronic kidney disease	10 (10.9%)	4 (13.8%)	0.74
Chronic liver disease	10 (10.9%)	3 (10.3%)	> 0.99
Data collected before NIV			
Respiratory rate, breaths/min	30 ± 7	28 ± 7	0.20
Heart rate, beats/min	121 ± 25	117 ± 26	0.39
Systolic blood pressure, mmHg	139 ± 31	135 ± 32	0.55
Diastolic blood pressure, mmHg	80 ± 18	78 ± 18	0.60
pH	7.22 ± 0.08	7.23 ± 0.08	0.66
PaCO_2_, mmHg	85 ± 23	82 ± 19	0.55
PaO_2_/FiO_2_, mmHg	192 ± 107	178 ± 77	0.50
GCS	12.6 ± 3.2	13.1 ± 2.8	0.47
Data collected at 1–2 h of NIV			
Respiratory rate, breaths/min	28 ± 7	26 ± 7	0.15
Heart rate, beats/min	117 ± 26	109 ± 24	0.11
Systolic blood pressure, mmHg	132 ± 34	125 ± 18	0.25
Diastolic blood pressure, mmHg	78 ± 19	74 ± 13	0.30
Minute ventilation, L/min	8.9 ± 4.1	9.8 ± 4.7	0.33
IPAP, cmH_2_O	17 ± 4	17 ± 5	0.89
EPAP, cmH_2_O	5 ± 2	6 ± 1	0.50
pH	7.21 ± 0.11	7.27 ± 0.09	< 0.01*
PaCO_2_, mmHg	90 ± 28	76 ± 20	0.02*
PaO_2_/FiO_2_, mmHg	164 ± 88	169 ± 64	0.79
GCS	11.5 ± 3.4	13.2 ± 2.0	0.01*
HACOR score before NIV	9.4 ± 3.7	8.9 ± 4.0	0.54
HACOR score at 1–2 h of NIV	13.9 ± 5.6	9.3 ± 3.4	< 0.01*
ICU mortality	33 (35.9%)	23 (79.3%)	< 0.01*
Hospital mortality	33 (35.9%)	23 (79.3%)	< 0.01*

*HACOR* heart rate, acidosis, consciousness, oxygenation and respiratory rate, *GCS* Glasgow coma scale, *NIV* noninvasive ventilation, *IPAP* inspiratory positive airway pressure, *EPAP* expiratory positive airway pressure, *ICU* intensive care unit

*p < 0.05 between two groups

## Discussion

This study developed a novel risk-scoring system, the HACOR score, for the prediction of NIV failure in COPD patients. This scale takes into account heart rate, acidosis, consciousness, oxygenation, and respiratory rate. Because the variables in the HACOR score are easily obtained using simple bedside measurements, it can serve as a rapid and convenient tool for predicting NIV failure. The HACOR score had good diagnostic accuracy for NIV failure when it was assessed at 1–2 h of NIV, and it was even better at predicting early NIV failure. In the high-risk patients identified by HACOR score, early intubation was associated with decreased hospital mortality.

It is advisable to attempt to predict NIV failure in COPD patients. However, multiple factors can cause NIV failure. Using a single variable, it is difficult to predict NIV failure with high accuracy. The combination of several variables can increase predictive power. Confalonieri et al. [5] reported a chart including APACHE II score, GCS, respiratory rate, and pH to predict NIV failure at initiation and 2 h of NIV in COPD patients. This chart had high accuracy in predicting NIV failure. However, the APACHE II score entails a large number of items and cannot be conveniently used to assess outcomes during an NIV intervention. Moreover, serum concentrations of creatinine and white blood cell counts are not always available within 1–2 h of NIV for every patient. Furthermore, no external validation was performed for that chart, and it remains unclear whether it can be extrapolated to other centers. Our scale (HACOR score) is simpler to apply to predict NIV failure in COPD patients. The HACOR score includes only heart rate, acidosis, consciousness, oxygenation, and respiratory rate for its prediction of NIV failure. Because these five variables are easily obtained at bedside, the HACOR score can be rapidly assessed. Furthermore, we performed internal and external validation of its predictive power. This increases the chance that the scale can be successfully extrapolated to other centers.

In COPD patients, NIV failure is highly associated with consciousness [22]. In our study, consciousness was the most relevant variable for predicting NIV failure, with a maximal score of 11 points. Arterial blood pH was the second most relevant variable, with a maximal score of 8 points. Heart rate, respiratory rate, and oxygenation were less relevant. Thus, the HACOR score reflects the different influences of these variables for the prediction of NIV failure. In addition, the sensitivity and specificity for predicting NIV failure were good at 1–2 h of NIV. As the HACOR score can accurately predict NIV failure in COPD patients and can easily be assessed at bedside, it can help clinical practitioners as they make the decision to continue or stop NIV.

The overall mortality was 23%–27% in patients with NIV failure [7, 8]. However, the mortality sharply increased (ranged from 50 to 80%) in patients with late NIV failure [12, 18, 23]. Therefore, identification of NIV failure and intubation earlier is a promising strategy to decrease hospital mortality. In our study, the HACOR score assessed at 1–2 h of NIV can identify the patients at high risk of NIV. Further, we performed a subgroup analysis and found that in these high-risk patients, early intubation was associated with decreased hospital mortality. Thus, the HACOR score is a potential tool for clinical physicians to identify NIV failure earlier. However, the high mortality in late failure group may be resulted from the high risk of mortality on baseline characteristics [18]. The clinical physicians should pay much attention on this issue when they use HACOR score to guide NIV management.

In a previous study, we showed that the HACOR score had high sensitivity and specificity for predicting NIV failure in patients with hypoxemic respiratory failure [19]. Although the variables for predicting NIV failure in that patient population are the same as in the COPD population, the weights for each variable are quite different. For example, although consciousness has the highest weight in both patient populations, oxygenation is the second most relevant variable in hypoxemic patients, and heart rate is the least relevant. These differences can be explained by the physiopathological differences between COPD and hypoxemic patients.

Among the three cohorts, we found an interesting result that patients in external-validation cohort not only had lowest pH and GCS but also had lowest NIV failure. However, they had lower proportion of underlying diseases, lower respiratory rate, lower heart rate, and lower APACHE II scores than those in other two cohorts. It may indicate that the population in external-validation cohort is different. This may explain that the predictive power in external-validation cohort is not as good as those in derivation and internal-validation cohorts. In addition, the IPAP was similar but EPAP was lower in external-validation cohort. It means that the patients in external-validation cohort received higher support pressure. Thus, the lower respiratory rate, lower heart rate, lower APACHE II score and higher support pressure contribute much to the lower NIV failure in external-validation cohort.

This study has several limitations. First, the internal-validation cohort was randomly selected. It may reduce the evidence strength. However, we divided the patients collected in the First Affiliated Hospital of Chongqing Medical University into four groups based on timeframe to internally validate the HACOR score once again. It still showed good predictive power of NIV failure in each group. Second, baseline state performance status and need for assistance with activities of daily living have been previously shown to be strongly associated with late NIV failure [18]. However, we did not collect these data which may have reduced the accuracy of the HACOR score to predict late NIV failure. Third, we did not predefine how the provision of care was performed in each hospital. The diversity in population and provision of care may be two reasons for different predictive power between the three cohorts. However, diversity is common between different hospitals and different populations. In the external-validation cohort, the predictive power reached moderate to high though it was lower than that in derivation and internal-validation cohorts. Moreover, the external validation only came from two hospitals. The generalizability was limited. Further validation of HACOR score in different settings is needed in the future. Fourth, we found that early intubation was associated with decreased mortality in high-risk patients identified by HACOR score assessed at 1–2 h of NIV. However, this is a secondary analysis. To confirm this result, prospective randomized controlled trial was encouraged.

## Conclusions

The HACOR score had high sensitivity and specificity for the prediction of NIV failure in COPD patients, particularly for early NIV failure. Higher scores indicate higher chances of NIV failure. As the variables for the HACOR score are easily obtained at bedside, it is convenient to use to assess the efficacy of NIV in COPD patients. In high-risk patients identified by HACOR score assessed at 1–2 h of NIV, early intubation is associated with decreased hospital mortality.

## Supplementary information

**Additional file 1: Figure S1.** How patients enrolled in each group.

**Additional file 2: Table S1.** Predictive power of NIV failure identified by HACOR score at 1–2 h of NIV among the patients hospitalized in the First Affiliated Hospital of Chongqing Medical University.

**Additional file 3: Table S2.** Comparisons in AUC among derivation, internal-validation, and external-validation cohorts.

**Additional file 4: Figure S2.** Comparison between HACOR score and risk chart developed by Confalonieri et al. to predict NIV failure in external-validation cohort [5].

**Additional file 5: Table S3.** Predictive power of NIV failure identified by HACOR score at 1–2 h of NIV.

**Additional file 6: Table S4.** Predictive power of early NIV failure identified by HACOR score at 1–2 h of NIV in overall cohorts.

**Additional file 7: Figure S3.** NIV decision tree developed by HACOR score.

## Data Availability

The datasets analyzed during the current study available from the corresponding author on reasonable request.

## Abbreviations

NIV: noninvasive ventilation
GCS: Glasgow coma scale
COPD: chronic obstructive pulmonary disease
IPAP: inspiratory positive airway pressure
EPAP: expiratory positive airway pressure
SE: sensitivity
SP: specificity
PPV: positive predictive value
NPV: negative predictive value
LR+: positive likelihood ratio
LR−: negative likelihood ratio
ICU: intensive care unit
OR: odds ratio
CI: confidence interval
AUC: area under the curve of receiver operating characteristics
HACOR: heart rate, acidosis, consciousness, oxygenation and respiratory rate
