# Supplementary figures and images for: Early prediction of noninvasive ventilation failure in COPD patients: derivation, internal validation, and external validation of a simple risk score
Source: Ann Intensive Care. 2019 Sep 30;9:108. doi: 10.1186/s13613-019-0585-9 (PMC6766459; doi:10.1186/s13613-019-0585-9)

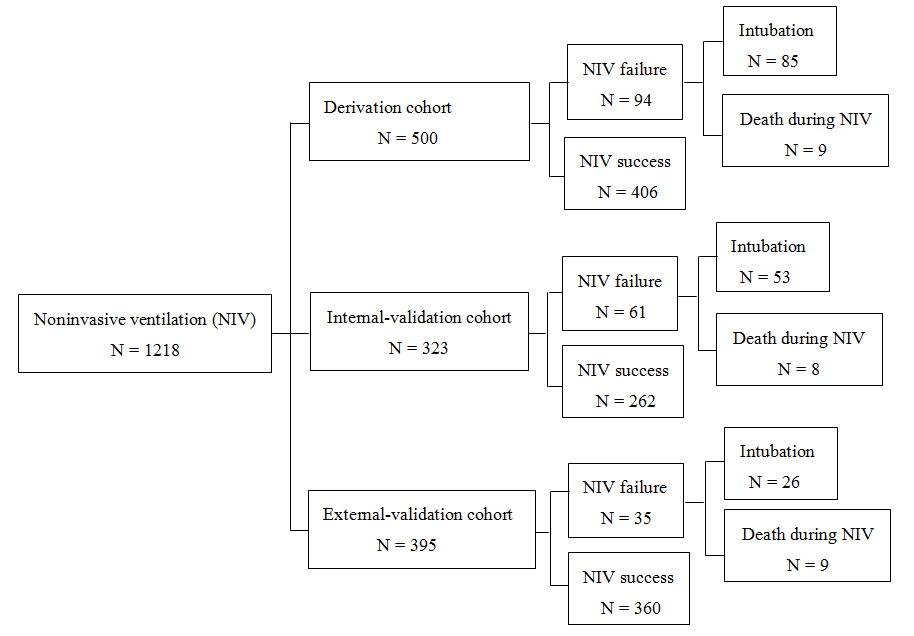

Supplement: Supplementary file 1 — Additional file 1: Figure S1. How patients enrolled in each group. [file 13613_2019_585_MOESM1_ESM.tif]

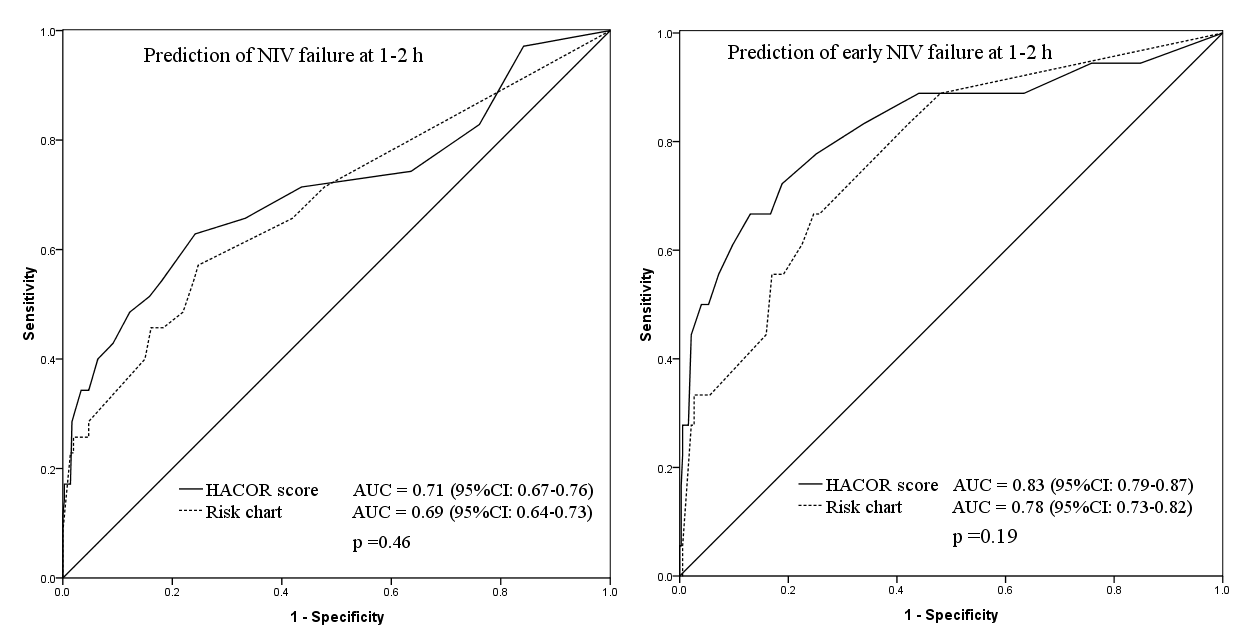

Supplement: Supplementary file 4 — Additional file 4: Figure S2. Comparison between HACOR score and risk chart developed by Confalonieri et al. to predict NIV failure in external-validation cohort [5]. [file 13613_2019_585_MOESM4_ESM.tif]

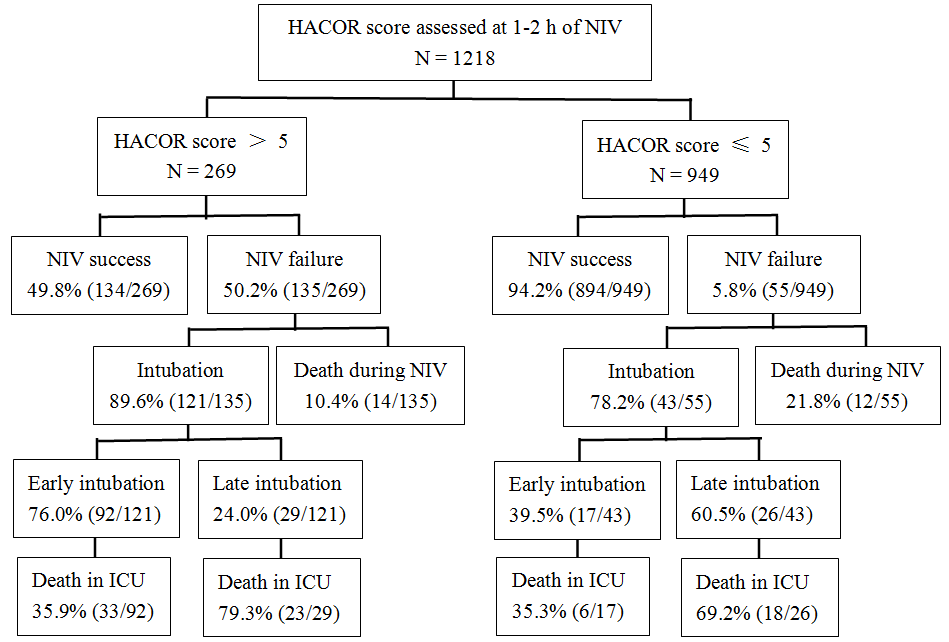

Supplement: Supplementary file 7 — Additional file 7: Figure S3. NIV decision tree developed by HACOR score. [file 13613_2019_585_MOESM7_ESM.tif]
