# Supplementary material for: Early prediction of noninvasive ventilation failure in COPD patients: derivation, internal validation, and external validation of a simple risk score
Source: Ann Intensive Care. 2019 Sep 30;9:108. doi: 10.1186/s13613-019-0585-9 (PMC6766459; doi:10.1186/s13613-019-0585-9)
Supplement: Supplementary file 2 — Additional file 2: Table S1. Predictive power of NIV failure identified by HACOR score at 1–2 h of NIV among the patients hospitalized in the First Affiliated Hospital of Chongqing Medical University. [file 13613_2019_585_MOESM2_ESM.doc]

Table S1. Predictive power of NIV failure identified by HACOR score at 1-2 h of NIV among the patients hospitalized in the First Affiliated Hospital of Chongqing Medical University.

| Subgroup | Cutoff point | AUC  (95%CI) | SE  (%) | SP  (%) | PPV  (%) | NPV  (%) | LR+ | LR- |
| --- | --- | --- | --- | --- | --- | --- | --- | --- |
| 2011-2012, N = 125 |  |  |  |  |  |  |  |  |
| Prediction of NIV failure | >5 | 0.87 (0.80–0.93) | 66.7% | 90.0% | 78.9% | 82.8% | 6.67 | 0.37 |
| Prediction of early NIV failure | >5 | 0.92 (0.86–0.96) | 87.5% | 83.2% | 55.3% | 96.6% | 5.20 | 0.15 |
| 2013-2014, N = 221 |  |  |  |  |  |  |  |  |
| Prediction of NIV failure | >5 | 0.89 (0.84–0.93) | 75.5% | 89.9% | 70.2% | 92.1% | 7.46 | 0.27 |
| Prediction of early NIV failure | >5 | 0.92 (0.87–0.95) | 83.8% | 85.9% | 55.4% | 96.3% | 5.93 | 0.19 |
| 2015-2016, N = 258 |  |  |  |  |  |  |  |  |
| Prediction of NIV failure | >5 | 0.93 (0.89–0.96) | 76.7% | 95.6% | 69.7% | 96.9% | 17.5 | 0.24 |
| Prediction of early NIV failure | >5 | 0.94 (0.90–0.97) | 89.5% | 93.3% | 51.5% | 99.1% | 13.4 | 0.11 |
| 2017-2018, N = 219 |  |  |  |  |  |  |  |  |
| Prediction of NIV failure | >5 | 0.86 (0.81–0.90) | 74.1% | 93.8% | 62.5% | 96.3% | 11.9 | 0.28 |
| Prediction of early NIV failure | >5 | 0.90 (0.86–0.94) | 82.4% | 91.1% | 43.8% | 98.4% | 9.24 | 0.19 |

HACOR = heart rate, acidosis, consciousness, oxygenation and respiratory rate, NIV = noninvasive ventilation, AUC = area under the curve of receiver operating characteristics, CI = confidence interval, SE = sensitivity, SP = specificity, PPV = positive predictive value, NPV = negative predictive value, LR+ = positive likelihood ratio, LR- = negative likelihood ratio.
