# Supplementary material for: Early prediction of noninvasive ventilation failure in COPD patients: derivation, internal validation, and external validation of a simple risk score
Source: Ann Intensive Care. 2019 Sep 30;9:108. doi: 10.1186/s13613-019-0585-9 (PMC6766459; doi:10.1186/s13613-019-0585-9)
Supplement: Supplementary file 3 — Additional file 3: Table S2. Comparisons in AUC among derivation, internal-validation, and external-validation cohorts. [file 13613_2019_585_MOESM3_ESM.doc]

Table S2. Comparisons in AUC among derivation, internal-validation, and external-validation cohorts

| Prediction of NIV failure | AUC (95%CI） |  | Prediction of early NIV failure | AUC (95%CI） |
| --- | --- | --- | --- | --- |
| Derivation cohort | 0.90 (0.87–0.92) |  | Derivation cohort | 0.91 (0.88–0.93) |
| Internal-validation cohort | 0.89 (0.85–0.92) |  | Internal-validation cohort | 0.96 (0.94–0.98) |
| External-validation cohort | 0.71 (0.67–0.76) |  | External-validation cohort | 0.83 (0.79–0.87) |
| *p^a^* | 0.72 |  | *p^a^* | 0.05 |
| *p^b^* | <0.01* |  | *p^b^* | 0.22 |
| *p^c^* | <0.01* |  | *p^c^* | 0.03* |

AUC = area under the curve of receiver operating characteristics, CI = confidence interval, NIV = noninvasive ventilation

*^a^P* denotes comparasions between derivation and internal-validation cohorts.

*^b^P* denotes comparasions between derivation and external-validationcohorts.

*^c^P* denotes comparasions between internal-validation and external-validation cohorts.

**p* < 0.05
