# Supplementary material for: Early prediction of noninvasive ventilation failure in COPD patients: derivation, internal validation, and external validation of a simple risk score
Source: Ann Intensive Care. 2019 Sep 30;9:108. doi: 10.1186/s13613-019-0585-9 (PMC6766459; doi:10.1186/s13613-019-0585-9)
Supplement: Supplementary file 5 — Additional file 5: Table S3. Predictive power of NIV failure identified by HACOR score at 1–2 h of NIV. [file 13613_2019_585_MOESM5_ESM.doc]

Table S3. Predictive power of NIV failure identified by HACOR score at 1-2 h of NIV.

| Cutoff point | SE  (%) | SP  (%) | PPV  (%) | NPV  (%) | LR+ | LR- |
| --- | --- | --- | --- | --- | --- | --- |
| Derivation cohort | |  |  |  |  |  |
| >0 | 98.9% | 21.2% | 22.5% | 98.9% | 1.26 | 0.05 |
| >1 | 94.7% | 33.5% | 24.8% | 96.5% | 1.42 | 0.16 |
| >2 | 92.6% | 59.1% | 34.4% | 97.2% | 2.26 | 0.13 |
| >3 | 87.2% | 74.6% | 44.3% | 96.2% | 3.44 | 0.17 |
| >4 | 80.9% | 87.9% | 60.8% | 95.2% | 6.70 | 0.22 |
| >5 | 70.2% | 92.6% | 68.7% | 93.1% | 9.50 | 0.32 |
| >6 | 62.8% | 96.3% | 79.8% | 91.8% | 17.0 | 0.39 |
| >7 | 55.3% | 98.0% | 86.7% | 90.5% | 28.1 | 0.46 |
| >8 | 53.2% | 99.0% | 92.6% | 90.1% | 54.0 | 0.47 |
| >9 | 44.7% | 99.0% | 91.3% | 88.5% | 45.4 | 0.56 |
| >10 | 37.2% | 99.3% | 92.1% | 87.2% | 50.4 | 0.63 |
| >11 | 31.9% | 99.3% | 90.9% | 86.3% | 43.2 | 0.69 |
| >12 | 27.7% | 99.8% | 96.2% | 85.6% | 112.3 | 0.73 |
| >13 | 21.3% | 100% | 100% | 84.7% | ∞ | 0.79 |
| Internal validation cohort | |  |  |  |  |  |
| >0 | 98.4% | 22.1% | 22.7% | 98.3% | 1.26 | 0.07 |
| >1 | 91.8% | 36.3% | 25.1% | 95.0% | 1.44 | 0.23 |
| >2 | 88.5% | 60.7% | 34.4% | 95.8% | 2.25 | 0.19 |
| >3 | 85.3% | 74.1% | 43.4% | 95.6% | 3.28 | 0.20 |
| >4 | 82.0% | 84.7% | 55.6% | 95.3% | 5.37 | 0.21 |
| >5 | 77.1% | 93.5% | 73.5% | 94.6% | 11.9 | 0.25 |
| >6 | 62.3% | 96.6% | 80.8% | 91.7% | 18.1 | 0.39 |
| >7 | 60.7% | 98.1% | 88.1% | 91.5% | 31.8 | 0.40 |
| >8 | 47.5% | 99.2% | 93.6% | 89.0% | 62.3 | 0.53 |
| >9 | 42.6% | 99.2% | 92.9% | 88.1% | 55.8 | 0.58 |
| >10 | 42.6% | 99.6% | 96.3% | 88.2% | 112 | 0.58 |
| >11 | 34.4% | 100% | 100% | 86.7% | ∞ | 0.66 |
| >12 | 32.8% | 100% | 100% | 86.5% | ∞ | 0.67 |
| >13 | 27.9% | 100% | 100% | 85.6% | ∞ | 0.72 |
| External validation cohort | |  |  |  |  |  |
| >0 | 97.1% | 15.8% | 10.1% | 98.3% | 1.15 | 0.18 |
| >1 | 82.9% | 23.9% | 9.6% | 93.4% | 1.09 | 0.72 |
| >2 | 74.3% | 36.4% | 10.2% | 93.5% | 1.17 | 0.71 |
| >3 | 71.4% | 56.4% | 13.8% | 95.3% | 1.64 | 0.51 |
| >4 | 65.7% | 66.7% | 16.1% | 95.2% | 1.97 | 0.51 |
| >5 | 62.9% | 75.8% | 20.2% | 95.4% | 2.60 | 0.49 |
| >6 | 54.3% | 81.9% | 22.7% | 94.8% | 3.01 | 0.56 |
| >7 | 51.4% | 84.2% | 24.0% | 94.7% | 3.25 | 0.58 |
| >8 | 48.6% | 87.8% | 28.0% | 94.6% | 3.97 | 0.59 |
| >9 | 42.9% | 90.8% | 31.3% | 94.2% | 4.68 | 0.63 |
| >10 | 40.0% | 93.6% | 37.9% | 94.1% | 6.26 | 0.64 |
| >11 | 34.3% | 95.3% | 41.5% | 93.7% | 7.26 | 0.69 |
| >12 | 34.3% | 95.8% | 44.5% | 93.7% | 8.23 | 0.69 |
| >13 | 34.3% | 96.7% | 50.1% | 93.8% | 8.23 | 0.69 |

HACOR = heart rate, acidosis, consciousness, oxygenation and respiratory rate, NIV = noninvasive ventilation, AUC = area under the curve of receiver operating characteristics, CI = confidence interval, SE = sensitivity, SP = specificity, PPV = positive predictive value, NPV = negative predictive value, LR+ = positive likelihood ratio, LR- = negative likelihood ratio.
