# Supplementary material for: Early prediction of noninvasive ventilation failure in COPD patients: derivation, internal validation, and external validation of a simple risk score
Source: Ann Intensive Care. 2019 Sep 30;9:108. doi: 10.1186/s13613-019-0585-9 (PMC6766459; doi:10.1186/s13613-019-0585-9)
Supplement: Supplementary file 6 — Additional file 6: Table S4. Predictive power of early NIV failure identified by HACOR score at 1–2 h of NIV in overall cohorts. [file 13613_2019_585_MOESM6_ESM.doc]

Table S4. Predictive power of early NIV failure identified by HACOR score at 1-2 h of NIV in overall cohorts.

| Cutoff point | SE  (%) | SP  (%) | PPV  (%) | NPV  (%) | LR+ | LR- |
| --- | --- | --- | --- | --- | --- | --- |
| >0 | 99.1% | 18.4% | 11.2% | 99.5% | 1.21 | 0.05 |
| >1 | 96.5% | 29.8% | 12.5% | 98.8% | 1.38 | 0.12 |
| >2 | 94.8% | 49.6% | 16.4% | 98.9% | 1.88 | 0.11 |
| >3 | 91.3% | 65.4% | 21.6% | 98.6% | 2.64 | 0.13 |
| >4 | 87.0% | 76.6% | 27.9% | 98.3% | 3.72 | 0.17 |
| >5 | 84.4% | 84.4% | 36.1% | 98.1% | 5.41 | 0.19 |
| >6 | 76.5% | 89.4% | 42.9% | 97.3% | 7.21 | 0.26 |
| >7 | 72.2% | 91.5% | 46.9% | 96.9% | 8.47 | 0.30 |
| >8 | 67.8% | 93.8% | 52.7% | 96.6% | 11.0 | 0.34 |
| >9 | 60.0% | 95.2% | 55.7% | 95.8% | 12.5 | 0.42 |
| >10 | 53.9% | 96.4% | 59.8% | 95.3% | 14.9 | 0.48 |
| >11 | 47.0% | 97.4% | 63.9% | 94.6% | 17.9 | 0.54 |
| >12 | 42.6% | 97.7% | 64.9% | 94.2% | 18.8 | 0.59 |
| >13 | 36.5% | 98.3% | 67.2% | 93.7% | 21.2 | 0.65 |

HACOR = heart rate, acidosis, consciousness, oxygenation and respiratory rate, NIV = noninvasive ventilation, AUC = area under the curve of receiver operating characteristics, CI = confidence interval, SE = sensitivity, SP = specificity, PPV = positive predictive value, NPV = negative predictive value, LR+ = positive likelihood ratio, LR- = negative likelihood ratio.
